# Isolation, Detection, and Quantification of Cancer Biomarkers in HPV-Associated Malignancies

**DOI:** 10.1038/s41598-017-02672-6

**Published:** 2017-06-12

**Authors:** Hakan Inan, Shuqi Wang, Fatih Inci, Murat Baday, Richard Zangar, Sailaja Kesiraju, Karen S. Anderson, Brian T. Cunningham, Utkan Demirci

**Affiliations:** 1 Demirci Bio-Acoustic-MEMS in Medicine (BAMM) Laboratory, Stanford University School of Medicine, Department of Radiology, Canary Center at Stanford for Cancer Early Detection, 3155 Porter Drive, Palo Alto, CA 94304 USA; 2 State Key Laboratory for Diagnosis and Treatment of Infectious Diseases, First Affiliated Hospital, College of Medicine, Zhejiang University, Hangzhou, China; 3 Pacific Northwest National Laboratory, Richland, WA USA; 4 Biodesign Institute, School of Life Sciences, Arizona State University, Tempe, AZ USA; 5 Department of Electrical and Computer Engineering, University of Illinois at Urbana-Champaign, Urbana, IL USA; 6 Department of Electrical Engineering (by courtesy), Stanford University, Stanford, CA USA

## Abstract

Human Papillomavirus (HPV) infection has been recognized as the main etiologic factor in the development of various cancers including penile, vulva, oropharyngeal and cervical cancers. In the development of cancer, persistent HPV infections induce E6 and E7 oncoproteins, which promote cell proliferation and carcinogenesis resulting elevated levels of host antibodies (*e.g*., anti-HPV16 E7 antibody). Currently, these cancers are clinically diagnosed using invasive biopsy-based tests, which are performed only in centralized labs by experienced clinical staff using time-consuming and expensive tools and technologies. Therefore, these obstacles constrain their utilization at primary care clinics and in remote settings, where resources are limited. Here, we present a rapid, inexpensive, reliable, easy-to-use, customized immunoassay platform following a microfluidic filter device to detect and quantify anti-HPV16 E7 antibodies from whole blood as a non-invasive assisting technology for diagnosis of HPV-associated malignancies, especially, at primary healthcare and remote settings. The platform can detect and quantify anti-HPV16 E7 antibody down to 2.87 ng/mL. We further validated our immunoassay in clinical patient samples and it provided significantly high responses as compared to control samples. Thus, it can be potentially implemented as a pretesting tool to identify high-risk groups for broad monitoring of HPV-associated cancers in resource-constrained settings.

## Introduction

Human Papillomavirus (HPV) infection is the most common sexually transmitted disease, and causes a variety of cancer types including penile, anus, vulva, oropharyngeal and cervical cancers^1^. Among these HPV-associated infections and malignancies, cervical and oropharyngeal cancers cause more than 300,000 mortalities in 2012 worldwide^2^. The most common oncogenic subtypes, HPV16 and HPV18, produce E6 and E7 oncoproteins^3^. Particularly, HPV16 E7 protein alone leads to cell immortalization, and promotes proliferation as well as inducing elevated levels of anti-HPV16 E7 antibody in sera^4^. In clinical practice, HPV-associated cancers are often diagnosed at late stages, since the disease progress asymptomatically, but antibody levels can predate overt cancer presentation by years^5^. Therefore, HPV16 E7 antibody has a potential as a blood-based biomarker for HPV-associated cancers^6^.

Currently, HPV-associated cancers are diagnosed using testing techniques including cytological examinations (Papanicolau test (Pap smear)), viral nucleic acid detection assays and tissue biopsy tests^7^. However, biopsy tissue and nucleic acid detection methods are invasive, require expensive reagents/equipment, skilled personnel, sophisticated and centralized laboratory infrastructure, as well as lengthy sample preprocessing and amplification steps^8^. In addition, Pap smear test has high false positive and false negative results due to operator readings^9^. Therefore, rapid, inexpensive, and easy-to-use pretesting assays for diagnosing of HPV-associated cancers to identify high-risk populations for further examination are urgently needed in environments with limited resources such as primary care and remote clinics.

Although, fluorescent bead-based assay (using Luminex^TM^ system)^4^ and programmable protein microarray tests^10^ have recently been used to quantify HPV16 E7 antibody from sera they still require significantly expensive devices, costly reagents and device maintenance as compared to conventional plate-based immunoassay tests as described in Table 1. In addition, these methods utilized blood samples with large volumes, which are processed using conventional centrifugation. However, centrifugation devices are not available at extreme poverty environments and cannot be used for blood separation at point-of-care (POC) applications.

**Table 1:** Techniques used for detection of HPV16 E7 antibody.

Technique	Device Cost	Multiplexing Capability	Availability at RCS	Blood collection at POC
Fluorescent bead-based assay^4^	High	Yes	Not Available	Not applicable
Programmable protein microarray^10^	High	Yes	Not Available	Not applicable
Our immunoassay platform with filter device	Low	Limited	Available	Applicable

Recent advances in microfluidics and nanotechnology-based biosensors hold great promise to address some of these challenges enabling integration of sensing technologies such as flat panel organic light emitting diodes with multiplex detection of cancer biomarkers^11^. However, these technologies face critical impediments in whole blood handling for biomarker detection at the resource-constrained settings^12, 13^. For instance, most detection platforms require intensive sample preparation steps including centrifugation of complex bodily fluids such as whole blood^14^. Currently, various blood separation approaches including membrane-based filters are performed for applications in resource-constrained settings. However, most of these platforms (i) require complex fabrication steps, (ii) rely on power-dependent active external fields (*e.g*., magnetic field, electrical field), and (iii) have low throughput separation processes (10 μL/hour). On the other hand, membrane-based filters possess various advantages including, low cost, easy-to-use, and ability to integrate with microfluidics platforms as summarized in Table 2.

**Table 2:** Comparison of filtration techniques for the separation of plasma from whole blood.

Technique	Example	Processing Volume	Material/Fabrication/ Availability	Applicability in RCSs and integration to Immunoassays
Density-based separation	Centrifuge^24^	>100 μL/min	Industrial device/ Commercial Product/Clinical Laboratory Research Laboratory	Requires for highly equipped laboratory, transfer of sample to center laboratories, expensive, a bench-top device, and need large sample volume to process
Geometry-based separation	Weir type^15^, Pillar type^15^, SIMBAS^25^	<10 μL/min	Si, SiO_2_, Polymer (*e.g*., PDMS, PMMA)/Optical Lithography /Research Laboratory	Requires highly expensive fabrication steps in clean room facilities, skilled personnel, not suitable for ELISA due to low volume yield, clogging issue
Flow-based separation	Continuous Flow^26^ Cross Flow^27^, Zweifach-Fung^28^	>10 μL /min >10 μL /min <10 μL /min	Si, SiO_2_, Polymer (*e.g*., PDMS, PMMA)/Optical Lithography / Research Labs	Requires highly expensive fabrication steps in clean room facilities.Not adequate for plasma separation
External Field-based separation	Dielectrophoresis^29^ Magnetophoresis ^30^ Acoustophoresis^31^	<1 μL/min	Si, SiO_2_,Polymer (*e.g*., PDMS, PMMA)/Optical Lithography/Research Labs	Depends on continuous external field, long operation time (*e.g*., 10 μL/ hour) High cost for fabrication steps (clean room)
Membrane-based separation	Porous filter membrane^16, 19, 32^	>10 μL/min	Polycarbonate/ Commercial Product/ Readily Deployable to RCSs	Inexpensive, easy to construct (plug-and-use), volume obtained is enough for Immunoassay tests e.g., ELISA

**Figure 1:**
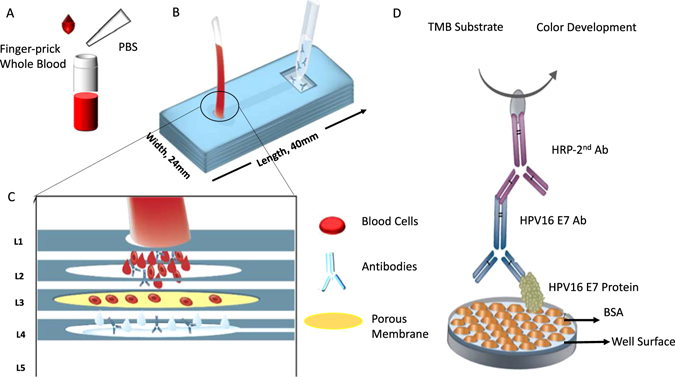
Design and construction of a small volume microfluidic device to selectively separate anti-HPV16 E7 antibodies and other proteins in plasma from the blood cells and their quantification by a customized immunoassay. (**A**) Finger prick volume of whole blood is collected and Initially only 15 µL whole blood was used and diluted with PBS in 1:20. (**B**) DSA layers were used to assemble the PMMA layers. After chip construction steps, blood sample was applied through the inlet, and filtrate was then collected from the reservoir. Approximately 100 µL of filtrate was collected. (**C**) A porous membrane is used to isolate the target antibodies from the whole blood by size-based filtration. Blood cells were retained on the membrane while plasma containing antibodies pass through. (**D**) Customized immunoassay was designed to detect anti-HPV16 E7 antibody from the filtered sample. Briefly, in the immunoassay, each well on 96-well plate was first coated with HPV16 E7 protein. Then, the filtered plasma sample containing anti-HPV16 E7 antibody (primary antibody) was applied to the wells. To generate a color development (blue to yellow), HRP-conjugated secondary antibody was coupled with TMB solution. The absorbance value at 450 nm was determined as the signal unit of customized immunoassay. Image courtesy of “Amy Thomas” for sub-figures (**B**,**C** and **D**).

Here, we present a non-invasive, inexpensive, rapid, and easy-to-perform customized immunoassay test with a filter-based microfluidic plasma separator for isolation and quantification of biomarkers (*i.e*., HPV16 E7 antibody) in determination of high risk groups for HPV-associated cancers at primary healthcare and remote clinics (Fig. 1) for subsequent testing. We further evaluated our immunoassay test with clinical samples using patient and healthy control plasma samples. We obtained a testing assay with receiver operative characteristic (ROC) curve indicating high reliability. This customized immunoassay test with a microfluidic filter device presents a rapid, reliable, and non-invasive pre-testing tool, and reduces the financial burden of current methods for broad monitoring of HPV-associated cancers in large populations in environments with limited resources. In addition, this platform has a great potential to obtain biotargets from a fingerprick volume of blood using a microfluidic filter device, thus potentially facilitating biosensing applications at resource-constrained settings.

## Materials and Methods

### Materials

Poly methyl methacrylate (PMMA) was purchased from McMaster-Carr (Atlanta, GA). Double-sided adhesive polymer film (DSA) was purchased from iTapestore (Scotch Plains, NJ). Millipore isopore polycarbonate membrane was obtained from Merck KGaA, (Darmstadt, Germany). Plastic tubing was purchased from Cole-Parmer (Vernon Hills, IL). Phosphate buffered saline (PBS) was obtained from Life Technologies (Grand Island, NY). Human IgG ELISA Kit was purchased from Abcam (ab100547) (Cambridge, MA). Whole Blood (EDTA) was obtained from Stanford Blood Center (Palo Alto, CA). 3,3′,5,5′-Tetramethylbenzidine (TMB substrate), and Bovine serum albumin powder (BSA) were purchased from Sigma Aldrich (St. Louis, MO). Peroxidase AffiniPure Sheep Anti-Mouse IgG (H + L) (HRP) and Peroxidase AffiniPure Goat Anti-Human IgG, Fcγ Fragment Specific was purchased from Jackson ImmunoResearch Laboratories, Inc. (West Grove, PA). BupH Carbonate-Bicarbonate buffer packs were obtained from Thermo Scientific (Rockford, IL). anti-HPV16 E7 antibody was purchased from Santa Cruz Biotechnology (sc-6981, Dallas, TX). HPV16 E7 protein was provided by Professor Karen Anderson, Arizona State University (Tempe, AZ).

### Methods

The experiment procedure and steps were indicated in seven sections. Briefly, (i) we fabricated the microfluidic filter device, (ii) set up the filtering process. (iii) Then, a commercial ELISA test kit (Abcam, ab100547) was used to evaluate the device performance as the proof of concept. (iv) Expression and Purification of HPV16 E7 Protein was performed. (v) We further developed a customized immunoassay test for detection and quantification of anti-HPV16 E7 antibody. (vi) We integrated this ELISA process with microfluidic filter device to isolate and quantify anti-HPV16 E7 antibody directly from whole blood. (vii) Lastly, we validated our platform with clinical samples obtained from OPC patients. These steps were detailed below in the following sections.

(i) **Fabrication of the microfluidic filter device**. The microfluidic filter device was constructed with poly methyl methacrylate (PMMA, 1.5 mm thickness), double-sided adhesive (DSA) film (50 μm thick), polycarbonate membrane (2 μm pore size)^11, 15^, and plastic tubing (0.025 mm in diameter). PMMA and DSA film were cut using a laser cutter system (Versa VLS2.3^TM^, Scottsdale, AZ) to prepare the layers. Briefly, the devices are built with five PMMA layers (PMMA-Layer 1 – PMMA-Layer 5), five DSA layers (DSA-1 – DSA-5) and one filter membrane (Fig. 1A). To allow blood filtration through these layers, we designed holes in each layer, where a circular hole was used for inlet and a square hole for the collection of filtered plasma. PMMA-Layer 1 was designed with a circular inlet (2 mm in diameter) and a square outlet (8 mm × 8 mm). Similarly, we designed PMMA-Layer 2–4 with a circular opening (6 mm in diameter) and a square opening (8 mm × 8 mm). Further, PMMA-Layer 4 was decorated with a 1.7 mm × 11 mm channel to connect inlet and outlet. Lastly, PMMA-Layer 5 was used as the base of device. The filter membrane was placed on the top of the circular opening in the PMMA-Layer 2. After all layers (PMMA and DSA) and membrane were assembled, the device presented one inlet and one outlet port. Blood sample was then applied through the inlet, passed through the filter, and filtrate was collected in the outlet reservoir (Fig. 1B).
(ii) **Filtering Process**. De-identified anonymous whole blood was purchased from Stanford Blood Center (Palo Alto, CA). We first diluted whole blood with phosphate buffered saline (PBS) (1:20 ratio) to prepare samples (final volume was 300 µL). To flow blood samples into microfluidic device, we then used a syringe pump (New Era Pumping System Inc., Farmingdale, NY). The flow rate and the membrane size was utilized on the basis of our recent work, in which we determined the optimum membrane size (2 μm) and flow rate (10 μL/minute)^16^. The flow rate was set at 10 μL/minute to separate plasma with protein content, and filtrate was collected at the output reservoir^16^. On top of the filter membrane, red blood cells (RBCs) and white blood cells (WBCs) were retained (Fig. 1B) while smaller molecules and plasma passed through. At the end of filtration process, we collected 100 μL of filtrated plasma for further immunoassay testing. The recovery rate of the biotargets was calculated by using the following equation;
  1 $${\rm{Recovery}}\,\,{\rm{Rate}}=({\rm{Biotarget}}\,{\rm{amount}}\,{\rm{from}}\,\text{device}/\text{Biotarget}\,{\rm{amount}}\,{\rm{from}}\,{\rm{centrifugation}})\times 100$$
(iii) **Evaluation of microfluidic filter device**. As a pilot study, we assessed the device performance to isolate IgG proteins from blood. Initial concentration of IgG was measured using a commercial Human IgG ELISA Kit (Abcam, ab100547, Cambridge, MA) as described in the manufacturer’s protocol. Here, we performed the filtration experiments with three different filter devices (n = 3). The collected filtrate was then diluted into 1:10^7^ with PBS to avoid over-saturation in the immunoassay procedure. To create a standard curve, we used concentrations of IgG ranging from 0.021 ng/mL to 15 ng/mL. The control (blank) was determined as a sample without IgG. For IgG immunoassay testing, 100 μL of standard solutions and samples filtered by microfluidic device (n = 3) were added into each well of a pre-coated 96-well plate and incubated on a shaker plate at 4 °C for overnight. The plates were then washed with a washing buffer, followed by a secondary antibody binding step (anti-human IgG detection antibody) for an hour at room temperature. Streptavidin-conjugated with HRP and TMB substrates were used for coloring process by incubating the plate for 45 and 30 minutes respectively at room temperature. Then, we terminated the reaction using a stop solution from Human IgG ELISA Kit (Abcam, ab100547, Cambridge, MA). We then scanned the plate using a plate reader (TECAN Infinite M1000, Morrisville, NC), and absorbance value at 450 nm was used as signal unit in the immunoassay.
(iv) **Expression and Purification of HPV16 E7 Protein**. Full length HPV16 E7 gene was transferred into Gateway compatible destination pCPD_nHalo vector (http://dnasu.asu.edu/DNASU/Home.jsp) from pDONR221 vector by recombination cloning. Expression plasmids were transformed into *E. coli* strain BL21DE3 and isolated colonies were grown in Luria broth (LB) media for 6–8 hours at 37 °C. The cultures were then diluted into MJ9 media and grown at 37 °C until OD600 of 0.6 was reached and followed by induction with 0.5 mM isopropyl β-D-1-thiogalactopyranoside (IPTG). After 21 h incubation at 18 °C, the cells were centrifuged at 5000 × g for 20 min at 4 °C, re-suspended pellets in lysis buffer (50 mM HEPES, 150 mM NaCl, pH 7.5, Octylphenoxy poly (ethyleneoxy) ethanol (IGEPAL) 0.01%, 1 mM DTT, 25 µg/ml Deoxy ribo nuclease (DNase), 2 mg/ml Lysozyme, 5 mM MgSO_4_, 100 µM Phenyl methyl sulfonyl fluoride (PMSF). The lysate was centrifuged at 5000 × g for 20 min at 4 °C and the supernatant was mixed with Halo Tag beads (Promega) and allowed to bind overnight at 4 °C. The beads were washed five times with Purification buffer (50 mM HEPES, 150 mM NaCl, pH7.5, 1 mM DTT, 2 mM Adenosine 5′-triphosphate (ATP) and 5 mM MgSO_4_). HPV16 E7 protein was eluted from halo tag beads by Halo Tobacco etch virus (TEV) protease (Promega). Bradford assay was used to quantitate the protein using bovine serum albumin (BSA) protein standard. Purity of HPV16 E7 protein was determined by Sodium dodecyl Sulfate (SDS) poly acrylamide gel electrophoresis (PAGE) (Figure S1).
(v) **Developing an immunoassay approach**. We used 96-well microtiter plate, “Nunc-Immuno^TM^ Maxisorp^TM^”, to develop an immunoassay process for anti-HPV16 E7 antibody. Initially, the well surface was coated with E7 protein by diluting it in bicarbonate (NaHCO_3_) buffer. Bicarbonate buffer was prepared by using commercial “BupH Carbonate-Bicarbonate buffer packs” and it was dissolved in 500 mL deionized water to have final pH of 9.4. E7 protein (400 μg/mL) was further diluted in bicarbonate buffer into samples to adjust the concentrations to 1 μg/mL, 200 ng/mL, 100 ng/mL, 50 ng/mL and 25 ng/mL and 12.5 ng/mL. We added 100 μL of each of these diluted solutions into the wells. Meanwhile, the standard solutions were prepared by using commercial anti-HPV16 E7 antibody (Santa Cruz Biotechnology, sc-6981, Dallas, TX). We tried different concentrations of proteins in each well to evaluate the best coating protein concentration against antibody. We added 100 μL of each of these solutions into wells and incubated at 4°C overnight. The next day, the plates were washed with washing solution, which was prepared by a ratio of 0.05% Tween-20 in PBS (PBST). To remove unbound E7 protein from the wells, we washed the plate with 200 μL of PBST and dried it with a paper towel. This step was repeated 5 times, and in the last one, PBST was incubated for one minute. Then, BSA (3%, dissolved in PBST) was used as a blocking agent, and 200 μL of BSA solution was added into each well, followed by a 90-minute incubation. We washed the wells with PBST, and added 100 μL of anti-HPV16 E7 antibody (200 μg/mL) samples into wells of a 96 well plate as triplicates. Final antibody concentrations ranged from 9 μg/mL to 2 pg/mL. The plate was shaken at room temperature for an hour and was washed with PBST after incubation. 100 μL of HRP (diluted 1:10^4^ in PBS to a final concentration of 80 ng/mL) was added into each well and incubated for 1 hour at room temperature while shaking (for patient samples, human specific HRP was used). After incubation, the plate was washed with PBST. Then 100 μL of TMB substrate was added into each well and plate was left in dark in shaking for color development for 15 minutes. We added 50 μL of stop reagent for TMB substrate to finalize the reaction and at this step color turned into yellow from blue (Fig. 1C). The absorbance at 450 nm was read for each well in a plate reader. (TECAN, infinite M1000, Morrisville, NC) Limit of detection (LoD) and limit of quantitation (LoQ) for the antibody were calculated by using the following equation^17^;
  2 $${\rm{LoD}}={\rm{Mean}}\,{\rm{of}}\,{\rm{Blank}}+(3\times {\rm{Standard}}\,{\rm{Deviation}})$$
  3 $${\rm{LoQ}}={\rm{Mean}}\,{\rm{of}}\,{\rm{Blank}}+(10\times {\rm{Standard}}\,{\rm{Deviation}})$$
(vi) **Integration of the immunoassay approach with microfluidic filter device for quantification of anti-HPV16 E7 antibody from whole blood**. We performed the immunoassay procedure for capture and quantification of anti-HPV16 E7 antibody directly from whole blood plasma, which was separated using microfluidic filter device. We purchased fresh whole blood from Stanford Blood Center, and these anonymous samples were collected in the tubes, which were pre-coated with EDTA. Here, we prepared three types of samples: (a) Positive Control (plasma with antibody from centrifugation): Initially 100 μL antibody was spiked into the plasma, which was obtained from centrifugation process. This spiked solution was then serially diluted with PBS to obtain samples with concentrations of 200 ng/mL, 150 ng/mL, 100 ng/mL, 50 ng/mL, 25 ng/mL, 12.5 ng/mL, 6.25 ng/mL, 3.1 ng/mL, and 1.5 ng/mL. (b) Negative Control (plasma without antibody from centrifugation): Whole blood centrifuged and plasma was obtained. This plasma was also serially diluted with PBS to get samples with no antibody (c) Actual samples: Antibody spiked whole blood samples to be processed through microfluidic filter device. These samples were used as actual samples that went through microfluidic filter device. The antibody concentration was fixed at 25 ng/ml in this step to evaluate the recovery efficiency of the devices after filtration process. 300 μL of this antibody-spiked diluted whole blood were run into each filter device with a syringe pump and tubing at a constant rate of 10 μL/minute (New Era Pump Systems, Farmingdale, NY). A total of 5 microfluidic filter devices were used. This blood samples were flowed into microfluidic filter devices and at the end of filtration process 100 μL of filtrate was collected from the reservoirs. We performed the customized immunoassay procedure as described in the subsection (iv) of the materials method of this manuscript. Here, triplicates of positive controls, negative controls and actual samples were used in the immunoassay process. Then, absorbance was read at 450 nm in the plate reader. The flow chart process is detailed in Figures S2 –S4.
(vii) **Evaluation of the immunoassay in clinical samples**. We evaluated our customized immunoassay test capability in clinical plasma samples (obtained from Professor Karen Anderson, Arizona State University, Tempe, AZ. IRB-36563)^6^. We performed the test using 20 healthy control samples and 18 OPC patient samples (Median age: 54, Male: 16, Female: 2). All the patients were diagnosed with OPC and HPV status was determined by p16 immunohistochemistry. The procedure described in “section v” was used. However, process was performed in biosafety laboratory (BSL2 + ) environment due to patient samples.

### Statistical Analysis

All statistical analyses were performed using Origin 8 (OriginLab Corporation, Northampton, MA-USA) and GraphPad Prism 7.00 (GraphPad Software, La Jolla California USA) softwares. We utilized one-way analysis of variance (ANOVA) followed by Tukey’s posthoc test (the significant threshold was set to 0.05, p < 0.05) and Mann-Whitney nonparametric analysis test (the significant threshold was set to 0.001, p < 0.001) for multiple comparisons of equal variances. Error bars represent standard deviations (SD) in all plots.

### Data Availability

All data generated or analyzed during this study are included in this published article (and its Supplementary Information files).

## Results and Discussion

### Assessing the performance of microfluidic filter device

**Figure 2:**
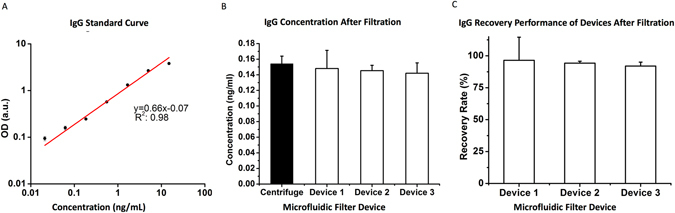
Evaluation of performance of microfluidic filter device using IgG proteins obtained directly from filtered whole blood. IgG in whole blood was used as a model biomarker to evaluate the performance of microfluidic device. Briefly, a commercial IgG ELISA test kit was used to detect the concentration of IgG from whole blood that was diluted 1:20 in PBS and filtered through device. As a control, same diluted blood sample was centrifuged (gold standard method) to obtain plasma. (**A**) A standard curve for IgG were generated using the standards in the commercial kit (n = 3). (**B**) After filtration via microfluidic device, the recovered IgG in whole blood was demonstrated in terms of concentration. Similarly, centrifugation results were presented in the plot. Concentrations were generated using the standard curve in Fig. 2A. There was no statistical difference between the control and microfluidic devices (n = 3, p > 0.05). (**C**) Recovery rate was calculated for microfluidic filter device. Centrifugation result was determined as 100% of recovery rate (gold standard). Average recovery rate obtained from three different microfluidic devices were calculated as 94% ± 2.0%. There is no statistical difference between the devices (n = 3, p > 0.05). Statistical analysis was performed using one way analysis of variance (ANOVA), followed by Tukey’s post hoc analysis (n = 3, p < 0.05).

In the experiments, we used IgG as a model biomarker, and compared the performance of microfluidic filter device with centrifugation (gold standard) method. For the quantification of IgG, we initially generated a standard curve using a commercial immunoassay (Table S1). In this curve, we worked with broad range of IgG concentrations between 0.021 ng/mL and 15 ng/mL (Fig. 2A). By utilizing this standard curve, we then quantified the IgG concentrations recovered from the microfluidic device and centrifugation method. In the microfluidic device, we used 15 μL of whole blood sample, which was diluted 1:20 ratio in PBS. The same blood samples were also used in the centrifugation method. As a result, the microfluidic filter devices recovered 145 ± 0.015 pg/mL of IgG (n = 9) after filtration process, whereas we obtained 154 ± 0.097 pg/mL of IgG in the centrifugation method (n = 3) (Fig. 2B). Here, we defined an equation, and calculated IgG recovery rate (Eq. 1). We calculated 94 ± 2% of IgG recovery for the microfluidic device using Eq. 1 (n = 3) (Fig. 2C).

### Evaluating the performance of customized immunoassay

**Figure 3:**
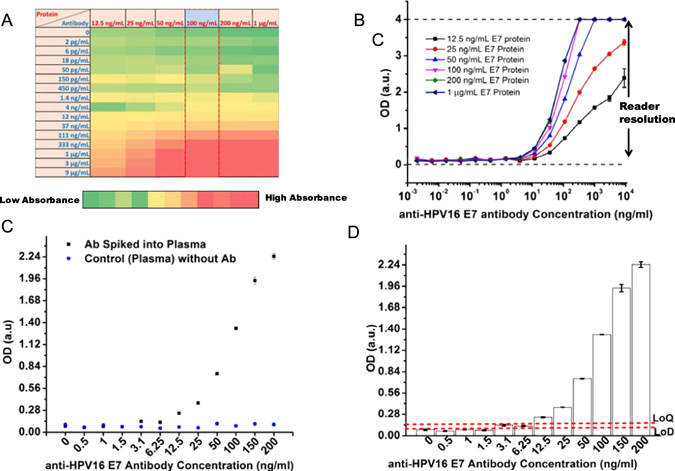
Developing the customized immunoassay by spiking anti-HPV16 E7 antibody into plasma and use various protein concentration. (**A**) In the experiments, we initially coated the wells surfaces with various concentrations of HPV16 E7 protein ranging from 12.5 ng/mL to 1 μg/mL. We then applied different concentrations of anti-HPV16 E7 antibody between 2 pg/mL and 9 μg/mL to these wells, followed by the color development using HRP-conjugated secondary antibody (80 ng/mL). The absorbance value at 450 nm was used as the signal unit of customized immunoassay. (**B**) As a result, we observed no differences in the OD values above 100 ng/mL of HPV16 E7 protein, which implied a saturation level for the protein. At the protein concentrations between 12.5 ng/mL and 100 ng/mL, we observed distinguishable OD levels. As manufacturer reported, the maximum resolution limit in absorbance (OD) of the plate reader is 4 a.u. Limit of detection (LoD) and limit of quantitation (LoQ) parameters of the customized immunoassay. In the experiments, we first spiked different concentrations of anti-HPV16 E7 antibody ranging from 1 ng/mL to 200 ng/mL into plasma. In this step, plasma samples were obtained using a centrifugation method. Negative control was defined as plasma sample without anti-HPV16 E7 antibody. Here, LoD and LoQ were used to evaluate immunoassay performance. (**C**) Experimental LoD was obtained using a statistical assessment by comparing OD values in plasma spiked samples and negative control. As a result, 3.1 ng/mL of anti-HPV16 E7 antibody concentration provided statistically different OD value compared to the negative control. (**D**) Theoretical LoD was calculated using Eq. 2. 2.87 ng/mL of anti-HPV16 E7 antibody concentration was calculated as theoretical LoD value and 6.5 ng/mL was calculated as LoQ value using Eq. 3. Statistical analyses were performed using one way analysis of variance (ANOVA), followed by Tukey’s post hoc analysis (n = 3, p < 0.05).

In the experiments, we used a 96-well format, indirect immunoassay procedure. We initially coated the wells surfaces with different HPV16 E7 protein concentrations ranging from 12.5 ng/mL to 1 μg/mL. We then applied various anti-HPV16 E7 antibody (primary antibody: target biomarker) concentrations ranging from 2 pg/mL to 9 μg/mL to these wells. These antibody concentrations were prepared with serial dilutions in PBS. For color development in the immunoassay, we used a fixed concentration of HRP-conjugated secondary antibody (80 ng/mL). In the experiments, the absorbance value at 450 nm was determined as the signal unit of customized immunoassay (Fig. 3A, Table S2). In the curve, we found no differences in the OD values above 100 ng/mL of HPV16 E7 protein (Fig. 3B and Figure S5A,B), which indicated a saturation level for the protein. Further, we observed distinguishable OD levels when we applied HPV16 E7 protein concentrations between 12.5 ng/mL and 100 ng/mL (Fig. 3B). In addition, the standard curves for these protein concentrations also provided high linearity with R^2^ values of 0.99 (Figure S5C). As an outcome of these observations, we continued to coat the well surfaces with 100 ng/mL of HPV16 E7 protein.

After optimizing HPV16 E7 protein concentration, we tested the performance of immunoassay in clinical matrices. First, we spiked various concentrations of anti-HPV16 E7 antibody ranging from 1 ng/mL to 200 ng/mL into plasma. Negative control was determined as plasma sample without anti-HPV16 E7 antibody (Table S3). As a performance parameter, we assessed the LoD and LoQ of anti-HPV16 E7 antibody spiked in plasma samples (Fig. 3C). Briefly, the LoD was classified into two groups: (i) Experimental LoD, which we statistically evaluated OD values in plasma spiked samples and negative control, and (ii) theoretical LoD, which we calculated using Eq. 2, and LoQ was calculated using Eq. 3. As a result, we observed that 3.1 ng/mL of anti-HPV16 E7 antibody concentration provided statistically greater OD value than that of negative control (Fig. 3C). In the theoretical LoD calculations, we calculated ~0.0994 of OD value using Eq. 2, which was corresponding to 2.87 ng/mL of anti-HPV16 E7 antibody concentration (Fig. 3D). As an outcome, we observed comparable LoD values (3.1 ng/mL and 2.87 ng/mL) in both calculations. We calculated the OD value of LoQ as 0.1495 that corresponds to 6.5 ng/mL antibody concentration.

### Integration of microfluidic filter device with the customized immunoassay

**Figure 4:**
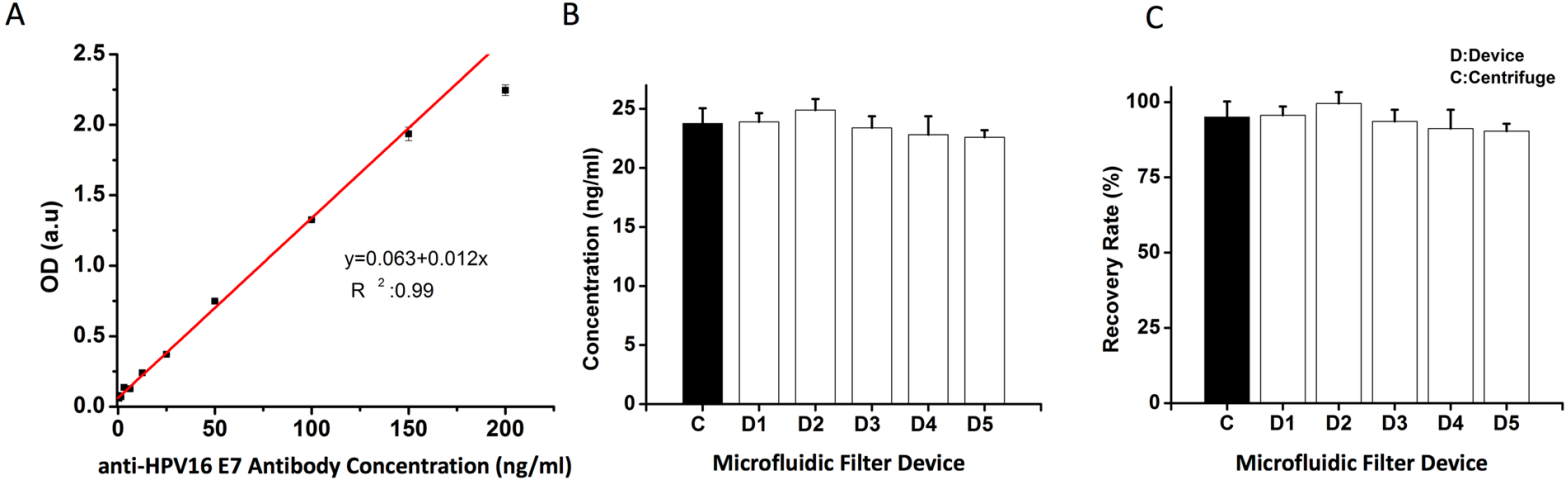
Integration of microfluidic filter device with the customized immunoassay using whole blood samples spiked with commercial HPV16 E7 antibodies. Here, we assessed the recovery rate of microfluidic filter device for detection of anti-HPV16 E7 antibody spiked whole blood, which was diluted 20 times before loading into the device. (**A**) A standard curve of immunoassay was generated using various concentrations of HPV16 E7 antibody spiked in plasma. (**B**) A fixed concentration of anti-HPV16 E7 antibody was spiked into whole blood, and then, the samples solution was prepared by diluting with PBS (1:20 ratio). The final concentration was adjusted to 25 ng/mL. By calculating the antibody concentrations using the standard curve, we observed that the centrifugation method recovered 23.76 ± 1.29 ng/mL of anti-HPV16 E7 antibody (n = 3), whereas the microfluidic device recovered 23.52 ± 0.92 ng/mL of anti-HPV16 E7 antibody (n = 3). As a result, no statistical difference was observed in between the microfluidic device and centrifugation method (n = 3, p > 0.05). (**C**) The antibody concentrations were converted to recovery rate using Eq. 1. As a result, 95 ± 5.17% and 94 ± 3.70% were observed for the centrifugation method and the microfluidic device, respectively. Statistical analyses were performed using one-way analysis of variance (ANOVA), followed by Tukey’s post hoc analysis (n = 3, p < 0.05).

Here, we first generated a standard curve of immunoassay using various concentrations of HPV16 E7 antibody spiked in plasma, and observed a high linearity in the curve with R^2^ values of 0.99 (Fig. 4A). This standard curve was used to quantify the recovered anti-HPV16 E7 antibody in the following experiment. Briefly, we first spiked a fixed concentration of anti-HPV16 E7 antibody into whole blood, and then, diluted this sample to 1:20 ratio in PBS (final concentration of 25 ng/mL) (Tables S4 and S6). To evaluate the performance of the microfluidic filter device, we calculated a recovery rate of anti-HPV16 E7 antibody, and compared the results of microfluidic device with the centrifugation method. By using the standard curve, we observed that the centrifugation method recovered 23.76 ± 1.29 ng/mL of anti-HPV16 E7 antibody (n = 3), whereas the microfluidic device recovered 23.52 ± 0.92 ng/mL of anti-HPV16 E7 antibody (n = 3). Further, there were no statistical differences observed in these two experimental sets (n = 3, p > 0.05) (Fig. 4B). In addition, we converted these antibody concentrations into recovery rate (Eq. 1). We calculated recovery rates as 95 ± 5.17% and 94 ± 3.70% for the centrifugation method and the microfluidic device, respectively (Fig. 4C). Overall, these observations indicated that our filter device provided comparable antibody recovery performance with the gold standard method (*i.e*., centrifugation method).

### Validation of customized immunoassay with clinical samples

**Figure 5:**
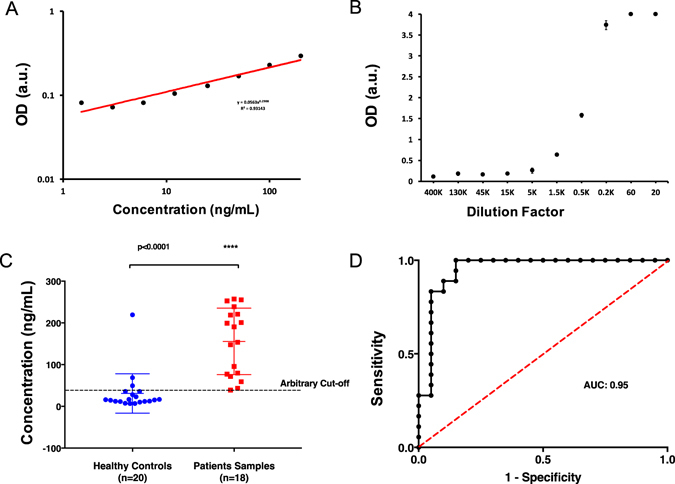
Validation of immunoassay test with clinical serum samples from 18 oropharyngeal cancer patients and 20 healthy controls. We performed our customized immunoassay test with clinical plasma samples from Oropharyngeal cancer patients. We have recorded the response of serially diluted patient sample (ID:02) in PBS. (**A**) Standard curve of the diluted patient sample. (**B**) Responses of varying dilutions (K: Thousand). Dilutions between 1.5 K and 0.2 K provided OD values within the linear range of the immunoassay. Therefore, 1:500 dilutions in PBS was used in the rest of the patient and healthy control samples. (**C**) We evaluated plasma samples from 20 healthy controls and 18 OPC patients. Patient samples have provided significantly high responses as compared to healthy controls. Controls have yielded false positive results too (particularly control ID:29). (**D**) We obtained ROC curve for our customized immunoassay test. Area under curve (AUC) of the system was 0.95 and the test had 94% sensitivity at 85% specificity. There was a significant difference between patient and control samples. Statistical analyses were performed using two-tailed Mann-Whitney test (n = 18, 20. P < 0.0001).

We evaluated the performance and the validity of our immunoassay test with 18 OPC patient serum samples and 20 healthy control samples (Table 3). To understand the response capacity of patients’ sample we initially obtained one sample (ID:7002) and serially diluted with PBS (from 1:20 to 1:400,000) and performed our immunoassay test with this sample. Among these, dilutions in between 1: 200 and 1: 1,500 were recorded within the linear region of the curve (Fig. 5A and B). Therefore, we used 1:500 dilutions as our dilution factor for the rest of clinical samples during immunoassay testing. We then, performed test for the remaining patient and controls samples. In general, patient samples provided significantly higher responses as compared to healthy controls (Fig. 5C). Further, our platform provided a reliable test results in distinguishing patient samples from healthy controls (Fig. 5D) at cut-off value of 38 ng/mL. Average healthy control response was measured at 31 ng/mL and patient sample response was at 162 ng/mL. Receiver operating characteristic (ROC) curve for the test was plotted and the area under the curve (AUC) was measured as 0.95. The sensitivity of test was 94% at 85% specificity. Average concentration of patient samples was calculated as 77 µg/mL by back-calculation for actual value in blood samples (Figure S6).

**Table 3:** Patient demographics.

Case IDs	Study Site	age(37–80)	gender	Tumor status	Tumor HPV status	P16
	Mount Sinai (MSSM),Oregon Health (OHSU)		1 = male, 2 = female	0 = negative 1 = positive	0 = negative 1 = positive	0 = negative 1 = positive
01	MSSM		1	1	1	1
02	MSSM		1	1	1	1
03	MSSM		1	1	1	1
04	MSSM		1	1	1	1
06	MSSM		1	1	1	1
08	MSSM		1	1	1	1
12	MSSM		2	1	1	1
13	MSSM		2	1	1	1
15	MSSM		1	1	1	1
16	MSSM		1	1	1	1
17	MSSM		1	1	1	1
18	MSSM		1	1	1	1
19	MSSM		1	1	1	1
20	MSSM		1	1	1	1
23	MSSM		1	1	1	1
26	MSSM		1	1	1	1
29	MSSM		1	1	1	1
32	MSSM		1	1	1	1
24	OHSU		2	0	0	0
25	OHSU		2	0	0	0
26	OHSU		2	0	0	0
27	OHSU		2	0	0	0
28	OHSU		2	0	0	0
29	OHSU		2	0	0	0
30	OHSU		1	0	0	0
31	OHSU		2	0	0	0
32	OHSU		2	0	0	0
34	OHSU		2	0	0	0
35	OHSU		2	0	0	0
36	OHSU		2	0	0	0
39	OHSU		2	0	0	0
40	OHSU		2	0	0	0
41	OHSU		2	0	0	0
42	OHSU		2	0	0	0
43	OHSU		2	0	0	0
44	OHSU		2	0	0	0
45	OHSU		2	0	0	0
46	OHSU		2	0	0	0

## Conclusion

HPV-associated cancers (including cervical cancer and OPC), are the leading causes of women deaths in developing world^2^. This problem is widely prevalent in the resource-constrained settings, where cancer diagnosis faces various challenges such as the need for expensive screening tools/assays, the lack of infrastructure and skilled personnel^18^. Although there are several POC-type platforms developed to detect and quantify blood-based cancer biomarkers, they face notable impediments including handling of whole blood and requirement of expensive devices and invasive techniques at resource-constrained settings, particularly in remote regions and in extreme poverty environments. (Table 1 and Table 2). To address these challenges in both technical and clinical aspects, we here utilized an immunoassay test combined with a microfluidic filter device. We developed an immunoassay test for the quantification of anti-HPV16 E7 antibody to identify high-risk groups for HPV-associated cancers and incorporated the assay with a microfluidic device for blood handling in extreme poverty environment. Microfluidic filter device is easy-to-use, inexpensive ($1.05 per device) (Table S7), and disposable that rapidly (20 min) separates blood plasma from finger prick volume (15 µL) of whole blood diluted 1:20 in PBS. We also validated our platform with clinical samples using healthy controls and OPC patient samples (Table 3) and obtained an accurate assay result. This immunoassay test in combination with the microfluidic filter device presents potentially a reliable platform for broad monitoring of HPV-associated cancers in large populations to identify high-risk groups for further examinations. Thus, providing a pretesting tool to reduce the financial, social, and technical challenges associated with current detection methods in resource-limited settings, particularly in remote regions of extreme poverty and in primary-care clinics, at developing countries. Further, the filter device can be utilized in extreme environments (such as remote villages where primary clinics are not present) for blood separation using a few drops of blood and collected plasma can be transported to primary clinics for immunoassay testing.

The microfluidic filter device consists of affordable materials, which are incorporated with filter to isolate plasma and biotargets from whole blood. PMMA layers and filter in the device are inexpensive substrates. The costs of materials used for the fabrication of device are provided in Table S7, these costs can be potentially reduced further with mass production. Automated fabrication of devices could further decrease variations in the standard errors of the measurements and could eliminate inter assay variations. Although membrane-based filters were used earlier to separate blood components^16, 19^, we here utilized the microfluidic filter device for separation of antibodies from whole blood targeting HPV-associated tumor detection. We also diluted the whole blood before filtration process to eliminate possible clogging issues that may result from blocking of filter membrane pores. This system allowed us to process whole blood in a single device with an active filter area of 28 mm^2^ millimeter squares. Although the blood volume that we processed was enough for us to perform the ELISA test, we anticipate that the processed whole blood capacity can be increased by using a larger filter device for other potential assays and applications. In future experiments, multiple filter membranes will be integrated into our microfluidic device for filtration process. Immunoassay test for quantification of HPV16 E7 antibody can be implemented in primary-care clinics with basic training and by using low-cost plate readers since fluorescent bead-based and programmable protein microarray tests are not available in these environments due to significant cost of devices. Further, this platform can be utilized for detection of other biomarkers from whole blood for different cancer models and diseases, especially for ear early cancer biomarkers. In the future, the microfluidic filter device can be potentially integrated into new generation platform technologies such as plasmonic^20^, nanomechanical^21^, and photonic crystal-based^22, 23^ approaches for isolation of other biotargets using unprocessed distinct bodily fluids at POC domain.

## Electronic supplementary material

Supplementary Information
