# Supplementary material for: Isolation, Detection, and Quantification of Cancer Biomarkers in HPV-Associated Malignancies
Source: Sci Rep. 2017 Jun 12;7:3322. doi: 10.1038/s41598-017-02672-6 (PMC5468352; doi:10.1038/s41598-017-02672-6)
Supplement: Supplementary file 1 — Supplementary Information [file 41598_2017_2672_MOESM1_ESM.pdf]

Supporting Information

Isolation, Detection, and Quantification of Cancer Biomarkers in HPV-Associated Malignancies

Hakan Inan[†] Shuqi Wang[‡], Fatih Inci[†], Murat Baday[†], Richard Zangar[¶], Sailaja Kesiraju[Δ], Karen S. Anderson[*,Δ], Brian T. Cunningham[*,⊥], Utkan Demirci[*,†, #]

[†] Demirci Bio-Acoustic-MEMS in Medicine (BAMM) Laboratory, Stanford University School of Medicine, Department of Radiology, Canary Center at Stanford for Cancer Early Detection, 3155 Porter Drive, Palo Alto, CA 94304, USA

[‡] State Key Laboratory for Diagnosis and Treatment of Infectious Diseases, First Affiliated Hospital, College of Medicine, Zhejiang University, Hangzhou, China

[¶] Pacific Northwest National Laboratory, Richland, WA, USA

[Δ] Biodesign Institute, School of Life Sciences, Arizona State University, Tempe, AZ, USA

[⊥] Department of Electrical and Computer Engineering, University of Illinois at Urbana-Champaign, Urbana, IL, USA

[#] Department of Electrical Engineering (by courtesy), Stanford University, Stanford, CA, USA

[*] Corresponding authors (Utkan Demirci, PhD, email: utkan@stanford.edu, Brian T. Cunningham, PhD, email: bcunning@illinois.edu, Karen S. Anderson, PhD (email: Karen.Anderson.1@asu.edu))

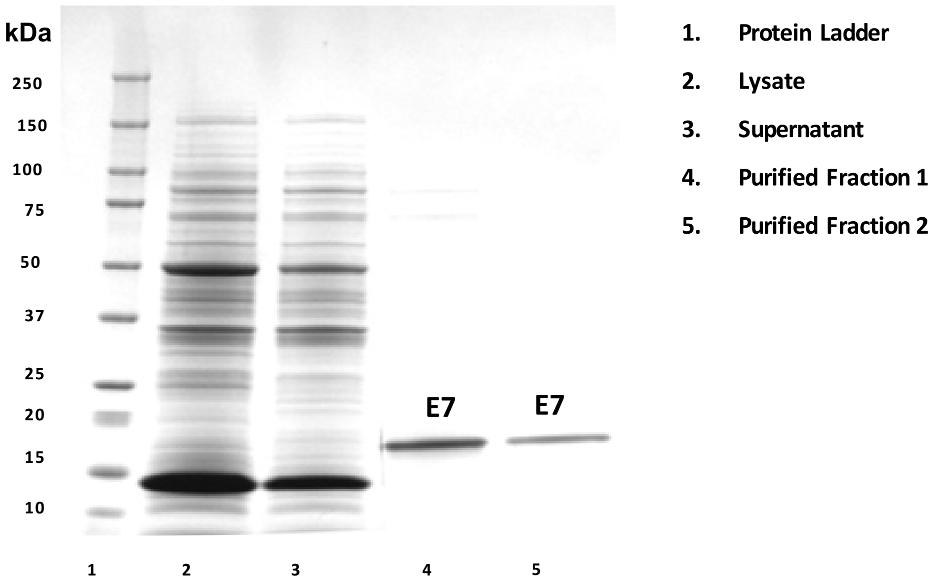

Fig. S1. SDS page Result of HPV16 E7 protein. Purity of protein is determined by Sodium Dodecyl Sulfate (SDS) poly acrylamide gel electrophoresis (PAGE).

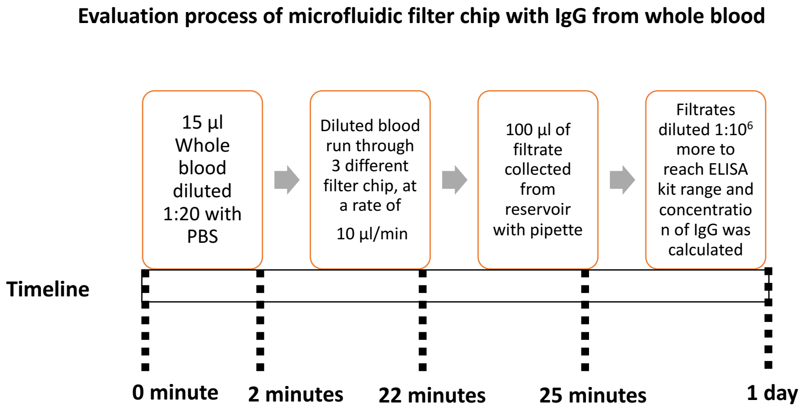

Fig. S2. Flow chart process of IgG evaluation step.

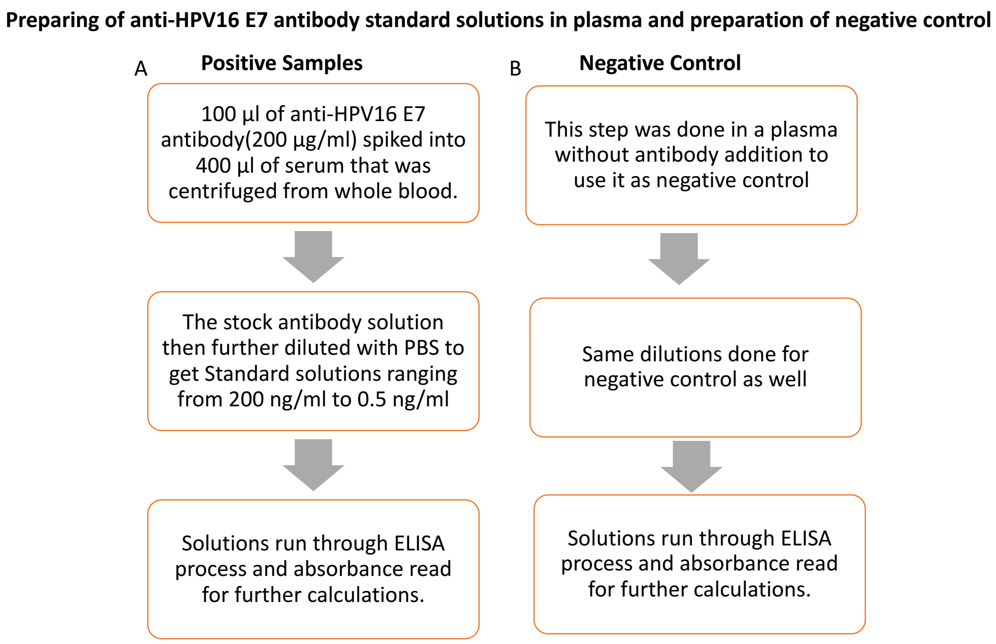

Fig. S3. Preparing of antibody with different concentrations to be used as standard solutions spiked in plasma. A) Positive control was prepared by spiking antibody into plasma and negative control was prepared by using plasma without antibody.

Filtering performance evaluation of antibody spiked whole blood through microfluidic filter chips

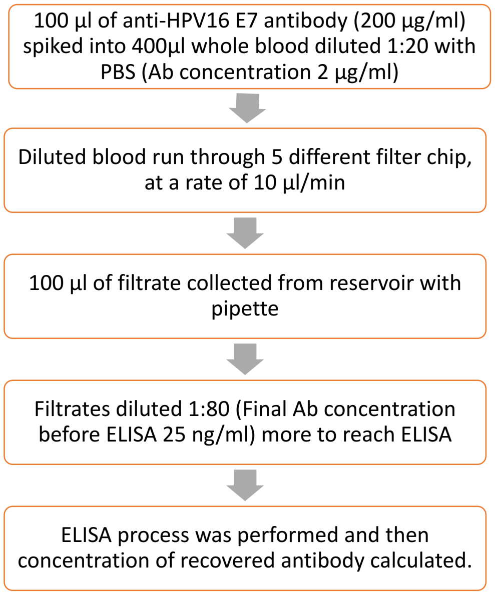

Fig. S4. anti-HPV16 E7 antibody recovery process after filtering through microfluidic filter device.

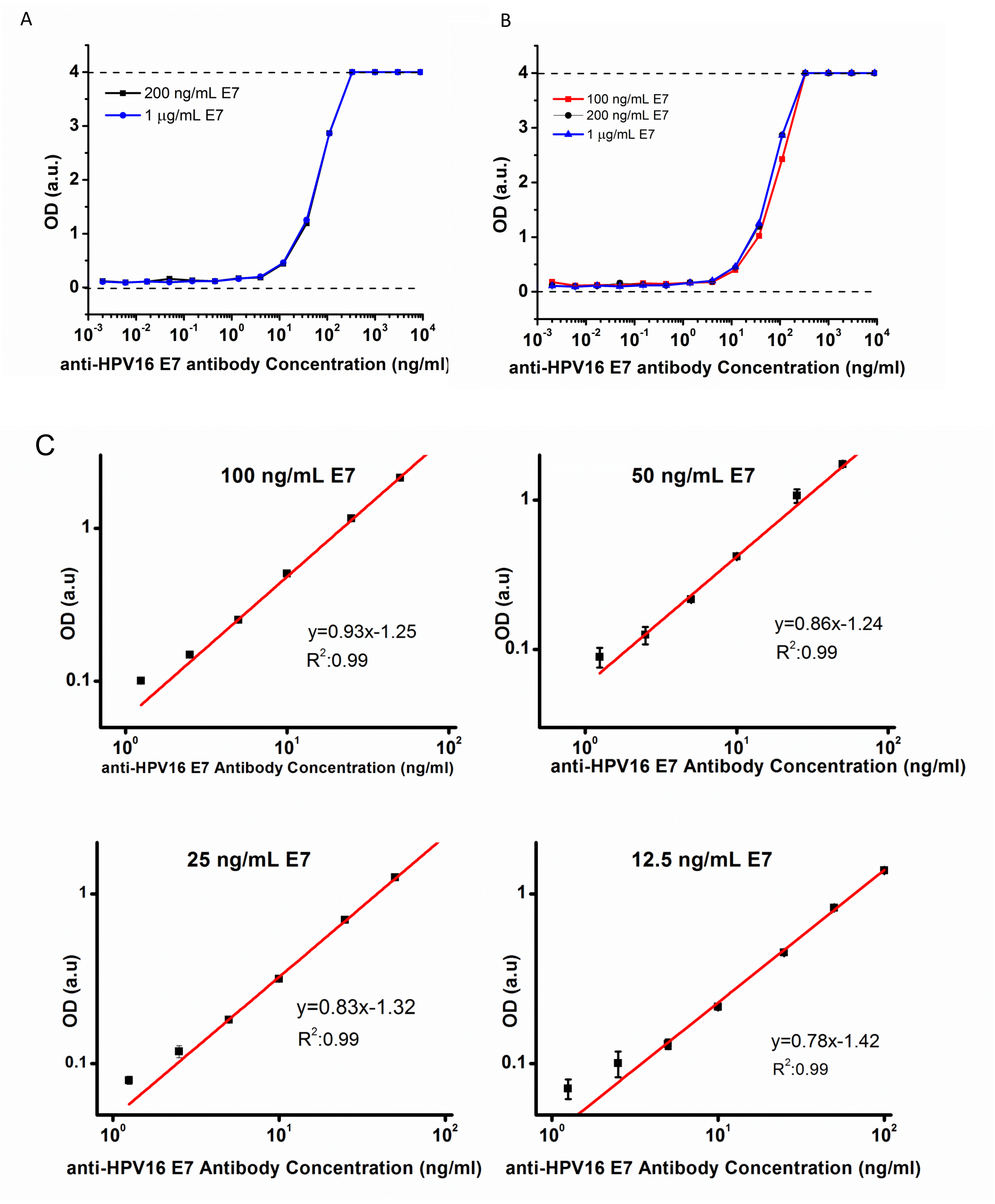

Fig. S5. Comparison of E7 protein coating in higher amounts. A. There is no absorbance value difference after 200 ng/mL coating. 1 µg/mL and 200 ng/mL have exactly the same value for all antibody concentrations. B. A difference started to appear

only after 100 ng/mL coating. C. Standard curve of protein coatings from 12.5 ng/mL to 100 ng/mL. All of the curves have R^2 value about 0.99. Further, 100 ng/mL was selected for further experiments since it produced higher OD values against low antibody concentrations which, would allow lower LoD capability.

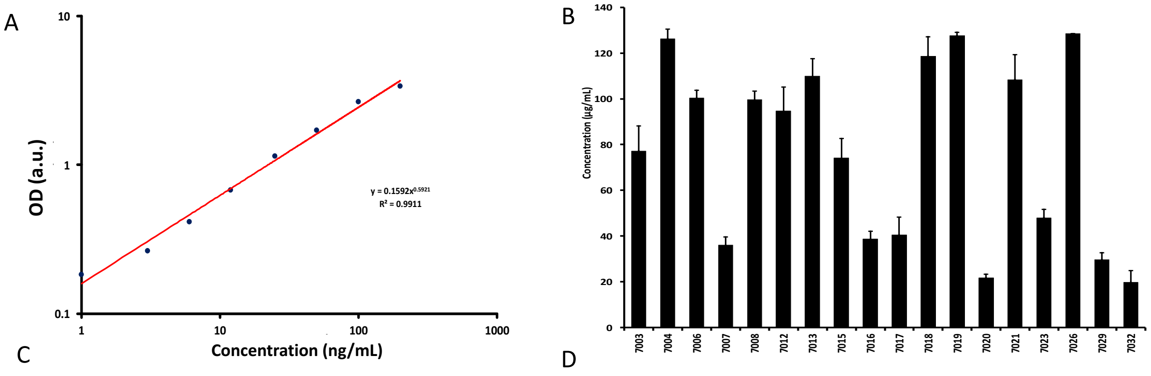

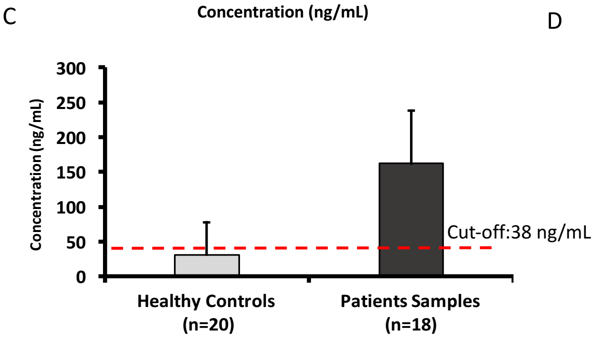

D

Average concentration comparison of clinical samples

Healthy Controls (n=20)	Patients Samples (n=18)
30.97946993	162.3533293

Fig. S6. A) Standard curve of the immunoassay test used to calculate the concentrations. B) HPV16 E7 antibody concentrations in patients' samples. We obtained high responses from patients' samples as compared to healthy controls. Average antibody actual concentration was 77.7 µg/mL found using back-calculation. C) Average response of healthy controls as compared to patients sample.

	Table S1: IgG Standard Curve				
**Standards**	**Concentration (ng/mL)**	**Abs 1**	**Abs 2**	**Mean**	**SE**
**s1**	15	3.8809	3.7276	3.80425	0.07665
**s2**	5	2.7267	2.6171	2.6719	0.0548
**s3**	1.667	1.3309	1.3076	1.31925	0.01165
**s4**	0.556	0.5791	0.5664	0.57275	0.00635
**s5**	0.185	0.2491	0.2424	0.24575	0.00335
**s6**	0.062	0.1691	0.1481	0.1586	0.0105
**s7**	0.021	0.1013	0.087	0.09415	0.00715

Table S2: ELISA Optimization of E7 Protein against anti-HPV16 E7 Antibody

Coated E7	Ab Concentration (ng/mL)	0	0.002	0.006	0.017	0.05	0.15	0.45	1.4	4	12	37	111	333	1000	3000	9000
	Well1	0.1308	0.1367	0.1244	0.1355	0.1293	0.1556	0.146	0.171	0.1042	0.1729	0.3339	0.7184	1.1744	1.5785	1.7733999	2.5716
	Well2	0.1146	0.1359	0.1252	0.1369	0.1392	0.1865	0.1328	0.1703	0.1086	0.1743	0.3355	0.7457	1.174	1.573	1.8832	2.2067001
	Mean	0.1227	0.1363	0.1248	0.1362	0.13425	0.17105	0.1394	0.17065	0.1064	0.1736	0.3347	0.73205	1.1742	1.57575	1.8283	2.38915
	SD	0.0114551	5.66E-04	5.66E-04	9.90E-04	0.0070004	0.0218496	0.0093338	4.95E-04	0.0031113	9.90E-04	0.0011314	0.019304	2.83E-04	0.0038891	0.0776404	0.2580232
12.5 ng/mL E7 Protein	SE	0.0081	4.00E-04	4.00E-04	7.00E-04	0.00495	0.01545	0.0066	3.50E-04	0.0022	7.00E-04	8.00E-04	0.01365	2.00E-04	0.00275	0.0549001	0.1824499
	Well1	0.1203	0.129	0.1243	0.1459	0.1394	0.164	0.1356	0.1749	0.1283	0.233	0.54	1.2047	1.9878	2.6343999	3.0609	3.3153999
	Well2	0.1258	0.1298	0.1191	0.1311	0.1362	0.162	0.1326	0.1565	0.1286	0.2277	0.5303	1.1693	2.0057001	2.6475	3.0335	3.4238
	Mean	0.12305	0.1294	0.1217	0.1385	0.1378	0.163	0.1341	0.1657	0.12845	0.23035	0.53515	1.187	1.9967501	2.64095	3.0472	3.3695999
	SD	0.0038891	5.66E-04	0.003677	0.0104652	0.0022627	0.0014142	0.0021213	0.0130108	2.12E-04	0.0037477	0.0068589	0.0250316	0.0126573	0.0092632	0.0193747	0.0766505
25 ng/mL E7 Protein	SE	0.00275	4.00E-04	0.0026	0.0074	0.0016	0.001	0.0015	0.0092	1.50E-04	0.00265	0.00485	0.0177	0.0089501	0.0065501	0.0137	0.0542001
	Well1	0.1139	0.1278	0.1137	0.1357	0.1466	0.1865	0.1338	0.1767	0.1575	0.3074	0.7883	1.7938	3.0552001	4	4	4
	Well2	0.0976	0.1247	0.1023	0.1292	0.1461	0.1786	0.1315	0.1692	0.1491	0.3236	0.8104	1.8193001	3.006	4	4	4
	Mean	0.10575	0.12625	0.108	0.13245	0.14635	0.18255	0.13265	0.17295	0.1533	0.3155	0.79935	1.80655	3.0306001	4	4	4
	SD	0.0115258	0.002192	0.008061	0.0045962	3.54E-04	0.0055861	0.0016263	0.0053033	0.0059397	0.0114551	0.0156271	0.0180313	0.0347897	0	0	0
50 ng/mL E7 Protein	SE	0.00815	0.00155	0.0057	0.00325	2.50E-04	0.00395	0.00115	0.00375	0.0042	0.0081	0.01105	0.01275	0.0246	0	0	0
	Well1	0.1108	0.1241	0.1147	0.1269	0.1289	0.1641	0.1721	0.1737	0.1816	0.4043	1.0321	2.4274001	4	4	4	4
	Well2	0.0982	0.2316	0.1064	0.1217	0.1111	0.145	0.1158	0.1605	0.1752	0.3925	1.0129	2.4219	4	4	4	4
	Mean	0.1045	0.17785	0.11055	0.1243	0.12	0.15455	0.14395	0.1671	0.1784	0.3984	1.0225	2.4246501	4	4	4	4
	SD	0.0089095	0.076014	0.005869	0.003677	0.0125865	0.0135057	0.0398101	0.0093338	0.0045255	0.0083439	0.0135764	0.0038891	0	0	0	0
100 ng/mL E7 Protein	SE	0.0063	0.05375	0.00415	0.0026	0.0089	0.00955	0.02815	0.0066	0.0032	0.0059	0.0096	0.00275	0	0	0	0
	Well1	0.0956	0.1097	0.0978	0.112	0.1124	0.1325	0.115	0.1714	0.1886	0.4503	1.1934	2.8388	4	4	4	4
	Well2	0.0888	0.125	0.0929	0.1052	0.2057	0.1283	0.1173	0.1706	0.1842	0.431	1.1928999	2.8945	4	4	4	4
	Mean	0.0922	0.11735	0.09535	0.1086	0.15905	0.1304	0.11615	0.171	0.1864	0.44065	1.19315	2.86665	4	4	4	4
	SD	0.0048083	0.0108187	0.0034648	0.0048083	0.0659731	0.0029698	0.0016263	5.66E-04	0.0031113	0.0136472	3.54E-04	0.0393859	0	0	0	0
200 ng/mL E7 Protein	SE	0.0034	0.00765	0.00245	0.0034	0.04665	0.0021	0.00115	4.00E-04	0.0022	0.00965	2.50E-04	0.02785	0	0	0	0
	Well1	0.0915	0.1113	0.0889	0.11	0.1022	0.1337	0.1263	0.1757	0.2021	0.4645	1.2639	2.8766	4	4	4	4
	Well2	0.088	0.1052	0.0926	0.1131	0.0937	0.1026	0.1078	0.1485	0.1979	0.4552	1.2417001	2.8462999	4	4	4	4
	Mean	0.08975	0.10825	0.09075	0.11155	0.09795	0.11815	0.11705	0.1621	0.2	0.45985	1.2528	2.86145	4	4	4	4
	SD	0.0024749	0.0043134	0.0026163	0.002192	0.0060104	0.021991	0.0130815	0.0192333	0.0029698	0.0065761	0.0156978	0.0214254	0	0	0	0
1 µg/mL E7 Protein	SE	0.00175	0.00305	0.00185	0.00155	0.00425	0.01555	0.00925	0.0136	0.0021	0.00465	0.0111	0.0151501	0	0	0	0

Table S3: anti-HPV16 E7 antibody LoD Calculation in Serum

Concentration	Standards (Positive Control)			Mean	SD	SE	Negative Control			Mean	SD	SE
0	0.0716	0.0857	0.0766	0.07797	0.00715	0.00413	0.1002	0.1011	0.0912	0.0975	0.00547	0.00316
0.5	0.0631	0.0588	0.0572	0.0597	0.00305	0.00176	0.0649	0.0669	0.0631	0.06497	0.0019	0.0011
1	0.0749	0.0759	0.0754	0.0754	5.00E-04	2.89E-04	0.0873	0.094	0.0894	0.09023	0.00343	0.00198
1.5	0.0687	0.0669	0.0766	0.07073	0.00516	0.00298	0.0712	0.0736	0.0652	0.07	0.00433	0.0025
3.1	0.1266	0.1403	0.145	0.1373	0.00956	0.00552	0.0752	0.0712	0.0661	0.07083	0.00456	0.00263
6.25	0.1264	0.1321	0.1226	0.12703	0.00478	0.00276	0.0497	0.0509	0.0512	0.0506	7.94E-04	4.58E-04
12.5	0.2419	0.2447	0.2357	0.24077	0.00461	0.00266	0.0666	0.0672	0.0679	0.06723	6.51E-04	3.76E-04
25	0.3741	0.3698	0.3706	0.3715	0.00229	0.00132	0.0638	0.0527	0.0537	0.05673	0.00614	0.00355
50	0.7508	0.7449	0.7494	0.74837	0.00308	0.00178	0.1012	0.1117	0.1122	0.10837	0.00621	0.00359
100	1.324	1.328	1.322	1.326	0.00283	0.002	0.081	0.0828	0.0793	0.08103	0.00175	0.00101
150	1.9695	1.902	1.93566	1.93575	0.04773	0.03375	0.1061	0.1162	0.0977	0.10667	0.00926	0.00535
200	2.2727	2.22	2.24567	2.24635	0.03726	0.02635	0.0969	0.1024	0.0941	0.0978	0.00422	0.00244

Table S4: Patients Samples

**Standard Curve**			
**Concentrations (ng/mL)**	**OD Values**		
200	3.3995	3.4061	3.2992
100	2.7108	2.6512	2.616
50	1.7872	1.7388	1.5989
25	1.174	1.1358	1.1257
12.5	0.68166	0.68031	0.67352
6.25	0.42018	0.41789	0.40942
3.125	0.26526	0.25874	0.27232
1.6	0.176	0.18517	0.18658
**Patients Samples**			
**Patient ID**	**OD Values**		
7003	3.2373	3.0056	3.014
7004	4	3.7859	4
7006	3.4066	3.4006	3.3211
7007	2.0912	2.2711	2.3349
7008	3.4295	3.3412	3.3265
7012	3.2073	3.2403	3.473
7013	3.3897	3.5713	3.6622
7015	3.1652	2.9627	3.0161
7016	2.4136	2.3573	2.195
7017	2.2227	2.6281	2.2437
7018	3.6718	3.9786	3.5584
7019	4	3.9239	3.9885
7020	1.5208	1.6826	1.6412
7021	3.3103	3.6006	3.6561
7023	2.5512	2.6907	2.5251
7026	4	4	4
7029	2.11	1.9912	1.8515
7032	1.7851	1.477	1.2403

Table S5: Healthy Control Samples			
**Standard Curve**			
**Concentrations (ng/mL)**	**OD Values**		
200	2.1639	2.4122	2.4073
100	1.5679	1.7959	1.7282
50	1.1804	1.2068	1.1933
25	0.74424	0.74594	0.77261
12.5	0.45717	0.4624	0.4604
6.25	0.29534	0.28483	0.28453
3.125	0.18753	0.18325	0.18057
1.6	0.12693	0.13351	0.13466
**Healthy Control Samples**			
**Contorl ID**	**OD Values**		
1524	0.26558	0.25039	0.26048
1525	0.36378	0.36173	0.37206
1526	0.2797	0.26262	0.27878
1527	0.33217	0.32616	0.35221
1528	1.0616	1.0475	1.0373
1529	2.9173	2.9366	2.9183
1530	0.24384	0.23977	0.23934
1531	0.45055	0.46303	0.47141
1532	0.37275	0.37297	0.37177
1534	0.24315	0.23716	0.25006
1535	0.38803	0.40583	0.41056
1536	0.6631	0.6657	0.69284
1539	0.32784	0.32843	0.356
1540	0.42246	0.4238	0.45017
1541	0.4517	0.45227	0.45815
1542	1.5032	1.5226	1.5159
1543	1.6677	1.65	1.6569
1544	0.90327	0.82819	0.77715
1545	0.74744	0.73087	0.7692
1546	0.48362	0.47299	0.50988
Blank	0.6618	0.6618	0.6618

Table S6. Concentration of antibody in different samples.

Sample	Standards	Positive Control	Negative Control	Actual Samples
**Process**	Ab spiked into E7 free plasma and diluted in PBS.	Ab spiked in whole blood diluted in PBS and centrifuged	Plasma from whole blood diluted in PBS.	Ab Spiked into whole blood, diluted in PBS run through microfluidic filter device
**Concentration**	0.5 ng/mL to 200 ng/mL	0.5 ng/mL to 200 ng/mL	0 ng/mL	25 ng/mL

Table S7 Cost table of a single microfluidic filter device.

	**Unit Price ($)**	**Quantity**	**Total ($)**
PMMA Layer	0.05	5	0.25
Filter Membrane	0.80	1	0.80
**Total Cost for a single microfluidic filter device**			**1.05**
